# Uniform Manifold Approximation and Projection Analysis of Soccer Players

**DOI:** 10.3390/e23070793

**Published:** 2021-06-23

**Authors:** António M. Lopes, José A. Tenreiro Machado

**Affiliations:** 1 INEGI, Faculty of Engineering, University of Porto, 4200-465 Porto, Portugal; 2 Institute of Engineering, Polytechnic of Porto, Dept. of Electrical Engineering, 4249-015 Porto, Portugal; jtm@isep.ipp.pt

**Keywords:** dimensionality reduction, clustering, data visualization, soccer, complex systems

## Abstract

In professional soccer, the choices made in forming a team lineup are crucial for achieving good results. Players are characterized by different skills and their relevance depends on the position that they occupy on the pitch. Experts can recognize similarities between players and their styles, but the procedures adopted are often subjective and prone to misclassification. The automatic recognition of players’ styles based on their diversity of skills can help coaches and technical directors to prepare a team for a competition, to substitute injured players during a season, or to hire players to fill gaps created by teammates that leave. The paper adopts dimensionality reduction, clustering and computer visualization tools to compare soccer players based on a set of attributes. The players are characterized by numerical vectors embedding their particular skills and these objects are then compared by means of suitable distances. The intermediate data is processed to generate meaningful representations of the original dataset according to the (dis)similarities between the objects. The results show that the adoption of dimensionality reduction, clustering and visualization tools for processing complex datasets is a key modeling option with current computational resources.

## 1. Introduction

Soccer is a complex system including multiple components that evolve at different scales both in time and in space. Presently, soccer has a huge economical and social relevance [1,2], but the study using advanced numerical and computational tools is still limited. We note that distinct levels of competition have been tackled, namely the technical progress of a player during his/her career [3,4,5], the time–space trajectories of the players in a match [6,7,8,9,10], or the performance of a number of teams along a league and season [11,12,13,14,15].

The prediction of the outcome of soccer matches is another important field, due to its interest both for the public, clubs, advertising companies, media and odds setters, besides researchers [16]. A variety of statistics tools have been adopted, namely Poisson models [17], Bayesian methods [18], rating systems [19] and machine learning schemes [20], among others [21,22].

The prediction of a match, league, or competition outcome is closely related to the concept of uncertainty. Uncertainty arouses fans’ emotion, is essential in the betting business, and is the factor that moves the sports industry. The uncertainty about the result of a match, a league, or any other competition, is measured by the ‘competitive balance’  [23,24]. In a league, or multi-team competition, the final standings of the teams is the main point of interest. If the competitiveness is high, then we have a high uncertainty in the match outcome, and vice versa, in what concerns the teams ranking in a league or competition [25]. Classical measures to quantify competitiveness either adopt simple ratios of standard features [26,27], or are developed based on graph theory [25].

Recent advances in the analysis of soccer dynamics have been accomplished with the developments registered in the area of sports analytics [28,29]. Sports analytics consists of the mathematical and statistical analysis of data related to sports, with the objective of providing a competitive advantage to a team or an individual. Often, we distinguish between on-field and off-field analytics [30]. The first deals with the improvement of the on-field behavior of players and teams, and, for example may address player fitness and game tactics. The second deals with business and focuses on helping sport organizations to increase ticket and merchandise sales, improve fans’ engagement and reach good management decisions, just to mention a few. Sports analytics developed rapidly in the last few years, supported by the technological advances in data measurement, storage and computational processing. Object-tracking tools allowed the automatic collection of information about players over time. The spatiotemporal datasets were adopted in a number of research works, including the retrieval of play sequences  [31] and the classification of defensive strategies [32] in basketball, and shot prediction [33] in tennis. Spatiotemporal data were used in soccer to identify play styles and team formations  [34], as well as to plan coordinated playing tactics [35].

The strategies to form competitive sports teams while having limited resources has attracted the attention of professionals, scientists and society. Scouting is fundamental in many sports, namely in professional soccer, to identify talented players [36]. Recognizing player styles and similarities between them are also crucial in forming a team lineup. To such purposes, scouts, technical directors and coaches often depend on heuristics (e.g., wage, specific abilities, previous experience and intuition) to choose players for their teams [37] independently of the time horizon of interest, that is, prior to, or during, a season or match. However, the standard adopted procedures are subjective and mistakes can lead to sport and economic failure. The rapid increase in the volume and quality of soccer digital data allowed for the application of computer tools to characterize and rank athletes under the light of their perceived abilities  [38]. Nonetheless, the automatic characterization of players based on such data is challenging in modern soccer [39], since players’ positions are not rigidly defined. Indeed, many players can occupy various roles on the field and each position requires a particular set of skills and physical attributes. Tools for searching relevant information in large soccer datasets motivated the interest of researchers in the field of computer science. Machine learning methods have been successfully applied in the prediction of match outcomes [20,40] and athletes’ injuries [41,42], analysis of team performance [43,44] and talent discovering [45,46], just to cite a few. The characterization and selection of players based on data is still a challenge.

The multidimensional nature of the data required to analyze soccer player styles and to compare elements between each other made the dimensionality reduction and clustering algorithms key tools to deal with soccer datasets. Dimensionality reduction-based schemes try to preserve in low dimensional representations the information embedded in the original datasets. They include linear methods, such as classic multidimensional scaling  [47], principal component [48], canonical correlation [49], linear discriminant [50] and factor analysis [51], as well as nonlinear approaches, such as non-classic MDS, or Sammon’s projection [52], isomap [53], Laplacian eigenmap [54], diffusion map [55], t-distributed stochastic neighbor embedding [56] and uniform manifold approximation and projection (UMAP) [57]. These techniques are closely connected to the field of information visualization, which corresponds to the computational generation of visual portraits of a dataset. Its main goal is to expose features embedded in the data, in order to understand the system that generated such data [58,59].

We find nowadays a vast literature on soccer data, but research based on dimensionality reduction, clustering and computer visualization of soccer players data is scarce. We can cite some works that adopt these techniques, although not necessarily all three together. Abade et al. [60] classified young players following their physical and physiological profiles gathered from training sessions in the point of view of age and playing position. The data from the time motion and the body acceleration/deceleration features were processed using repeated-measures factorial ANOVA and two-step cluster analysis to classify players. Fortuna et al. [61] analyzed the notoriety and international popularity of players in the viewpoint of Google queries over time. The data streams were processed through K-means clustering and three semi-metrics using the functional principal component decomposition and their first and second derivatives. Kirschstein and Liebscher [62] studied the athletes’ market value versus their performance skills by applying principal component analysis. Gavião et al. [63] used ranking, classification, dynamic evaluation and regularity analysis within the framework of composition of probabilistic preferences to determine the best investment opportunities when choosing among players.

This paper adopts dimensionality reduction, clustering and computer visualization tools to compare soccer players based on a set of attributes. The players are characterized by numerical data that rate their specific skills. The dataset used is retrieved from the soccer video game FIFA by Electronic Arts (EA) (https://www.ea.com/, accessed on 12 February 2021), which comprises realistic data about about 18,000 players worldwide. The players are viewed as objects that are compared by means of metrics that generate proper inputs to a UMAP algorithm. The UMAP produces meaningful representations of the original dataset according to the (dis)similarities between the objects. The results show that the adoption of dimensionality reduction and visualization tools for processing complex data is a key modeling option with current computational resources.

The paper structure is as follows. Section 2 and Section 3 introduce the UMAP algorithm, used for processing and visualizing the dataset, and the FIFA dataset, respectively. Section 4 analyses the data in a global perspective and interprets the results in the light of the geometric patterns generated. Section 5 compares the players based on their skills according to their position on the pitch. Section 6 presents the conclusions.

## 2. The Uniform Manifold Approximation and Projection

The UMAP is novel technique [57] for dimensionality reduction, clustering and visualization of high-dimensional datasets, which seeks to accurately represent both the local and global structures that characterize the information [64,65].

Let us consider a set of *N* objects, $v_{i}$, i=1,…,N, in a *r*-dimensional space. Those are represented in a *s*-dimensional embedding space, r≤s, by $t_{i}$, while preserving as best as possible the inter-object distances.

The UMAP computational tool requires a distance, $d(v_{i},v_{j})$, between pairs of objects $v_{i}$ and $v_{j}$, i,j=1,…,N, and the number of neighbors to consider, *k*. The algorithm has two main stages. In the first, it starts by computing the *k*-nearest neighbors of $v_{i}$, $N_{i}$, with respect to the distance $d(v_{i},v_{j})$. Then, the UMAP calculates the parameters $\rho_{i}$ and $\sigma_{i}$ for each data point $v_{i}$. The parameter $\rho_{i}$ stands for a nonzero distance between $v_{i}$ and its nearest neighbor and is determined as:

(1) $$\rho_{i}=\min_{j\in N_{i}}\left\{d\left(v_{i},v_{j}\right)|d\left(v_{i},v_{j}\right)>0\right\}.$$

The parameter $\rho_{i}$ plays a key role for assuring the local connectivity of the manifold. This means that $\rho_{i}$ yields a locally adaptive exponential kernel for each point.

The constant $\sigma_{i}$ must be chosen so that the following condition is satisfied:

(2) $$\log_{2}k=\sum_{j\in N_{i}}\exp[\frac{-\max(0,d\left(v_{i},v_{j}\right)-\rho_{i})}{\sigma_{i}}]$$

and it is determined using a binary search.

The algorithm determines a joint probability distribution $p_{ij}$ that measures the similarity between $v_{i}$ and $v_{j}$, in such a way that similar (dissimilar) objects are assigned a higher (lower) probability:

(3) $$p_{ij}=p_{j|i}+p_{i|j}-p_{j|i}p_{i|j},$$

(4) $$p_{j|i}=\{\begin{array}{cc} \exp[\frac{-\max(0,d\left(v_{i},v_{j}\right)-\rho_{i})}{\sigma_{i}}], & j\neq i\\ 0, & j=i \end{array},$$

where $p_{ij}=p_{ji}$, $p_{ii}=0$, $\sum_{i,j}p_{ij}=1$ and $\sum_{j}p_{j|i}=1,\forall i,j$.

In the second stage, the UMAP algorithm calculates the similarities between each pair of points in the embedding *s*-dimensional space:

(5) $$q_{ij}=q_{j|i}+q_{i|j}-q_{j|i}q_{i|j},$$

(6) $$q_{ij}=\{\begin{array}{cc} {\left[1+a||t_{i}-t_{j}{\left|\right|}^{2b}\right]}^{-1}, & j\neq i\\ 0, & j=i \end{array},$$

where $q_{ij}=q_{ji}$, $q_{ii}=0$, $\sum_{i,j}q_{ij}=1$ and $\sum_{j}q_{j|i}=1,\forall i,j$. The parameters *a* and *b* are either user-defined, or are determined by the algorithm given the required separation between close points, δ, in the embedding space:

(7) $${\left[1+a||t_{i}-t_{j}{\left|\right|}^{2b}\right]}^{-1}\approx \{\begin{array}{cc} 1, & t_{i}-t_{j}\leq \delta \\ \exp[-\left(t_{i}-t_{j}\right)-\delta ], & t_{i}-t_{j}>\delta \end{array}.$$

The UMAP performs an optimization, while minimizing the cross-entropy CE between the distribution of points in the original and the embedding spaces:

(8) $$CE=\sum_{i\neq j}[p_{ij}\ln\frac{p_{ij}}{q_{ij}}-\left(1-p_{ij}\right)\ln\frac{1-p_{ij}}{1-q_{ij}}].$$

The minimization procedure starts with a given initial set of points in the embedding space. The UMAP uses the Graph Laplacian to assign initial low-dimensional coordinates and, then, proceeds with the optimization using the gradient descent:

(9) $$\frac{\partial CE}{\partial t_{i}}=\sum_{j}[\frac{2ab{\left[d\left(t_{i},t_{j}\right)\right]}^{2(b-1)}}{1+a{\left[d\left(t_{i},t_{j}\right)\right]}^{2b}}p_{ij}-\frac{2b}{{\left[d\left(t_{i},t_{j}\right)\right]}^{2}\left(1+a{\left[d\left(t_{i},t_{j}\right)\right]}^{2b}\right)}\left(1-p_{ij}\right)]\left(t_{i}-t_{j}\right).$$

## 3. Description of the Dataset

Comprehensive datasets of sports are either obtained by the end-user through dedicated hardware and software tools, or are bought from professional service providers. Soccer-related statistics characterize specific aspects of the teams and players during a match, such as the percentage of time with ball possession, the number of attempts to goal and the number of finishes and turnovers. Moreover, we can also have, for a given season, the accumulated points, the average number of goals scored and suffered per match, and the average time to score, just to cite a few. These data are generated automatically by means of sensors, such as video cameras and 3D tracking motions systems, processed using specific software and organized in databases. Therefore, gathering such rich information about teams and players is costly and, therefore, has been available only to entities with high financial resources.

Fortunately, public sports-related datasets, ranging from individual players’ performance attributes and game statistics, to event logs of matches, have also became available to the scientific community and professionals. Concerning data about soccer players’ skills, besides those obtained using automatic procedures, knowledge comes also from coaches, former players, journalists and other sports agents. The precise characterization of players will allow a better understanding of teams, matches and leagues, as well as to improve the economic aspects of the modern soccer industry.

In this paper we use data from the FIFA 2021 video game. The FIFA was launched in 1995 by the company EA https://www.ea.com/ (accessed on 12 February 2021)and had new releases every year since. The EA provides an extensive database of soccer players. The players are assigned to five main groups based on their position on the pitch, as summarized in Table 1, and are characterized by a comprehensive set of attributes, both qualitative and quantitative. These attributes are gathered, curated and updated on a regular basis to reflect the real-life performances of the players. This task is carried out by professionals whose job is to bring the game as close to reality as possible, hence preserving coherence and representativeness across the dataset. Table 2 summarizes the most important subset of attributes adopted to characterize the two most popular players of the last decade: L. Messi and Cristiano Ronaldo (the names of all players are those adopted by the EA). For example, the sofifa_id is the unique code that identifies the player in the EA database. The overall, rated on a 0 to 100 scale, measures the quality of the player using a single numerical value calculated as a weighted sum of some attributes, namely those with number k=1,…,34. The potential, also rated on a 0 to 100 scale, measures the margin of progression that is expected for the player, based on his actual skills, age and some additional factors. The player_positions corresponds to, at least, one of those positions shown in Table 1, being that each player can have up to three positions assigned. The international_reputation, rated in the interval 1 to 5, takes into account the notoriety and the past carrier of the player. The attributes k=1,…,34 stand for the player skills and are rated on a 0 to 100 scale [66]. The data are available on the website www.sofifa.com (accessed on 12 February 2021) and can be viewed for one player at a time. Therefore, in this paper we use the data scraped from www.sofifa.com (accessed on 12 February 2021), available at the website https://www.kaggle.com/stefanoleone992/fifa-21-complete-player-dataset (accessed on 12 February 2021). The information is provided in csv format, one file per year, covering the period from 2015 up to 2021.

The FIFA 2021 raw dataset contains 18,944 players. However, after data cleaning for eliminating entries with missing or inaccurate values, we obtain a total of 18,708 players, distributed within the groups {Goalkeepers, Defenders, Centre Midfielders, Wingers, Strikers}, comprising {2054,6725,3556,2854,3519} athletes, as shown in Table 1.

**Figure 1:**
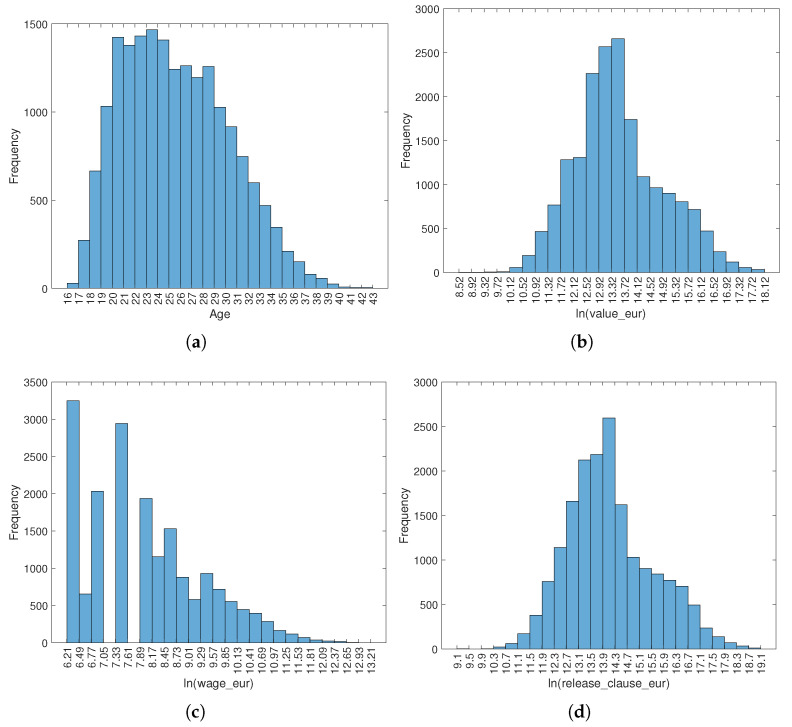
Histograms characterizing the FIFA 2021 dataset according to the attributes: (**a**) age; (**b**) ln(value_eur); (**c**) ln(wage_eur); (**d**) ln(release_clause_eur).

Figure 1 depicts the histograms that characterize the distributions of the players’ attributes age and the logarithm of value_eur, wage_eur and release_clause_eur. The log-transform of the numerical values for the attributes that have large variability is adopted to improve their visualization. We verify that age and ln(wage_eur) are moderately and highly right-skewed, respectively, while ln(value_eur) and ln(release_clause_eur) are almost similar.

**Figure 2:**
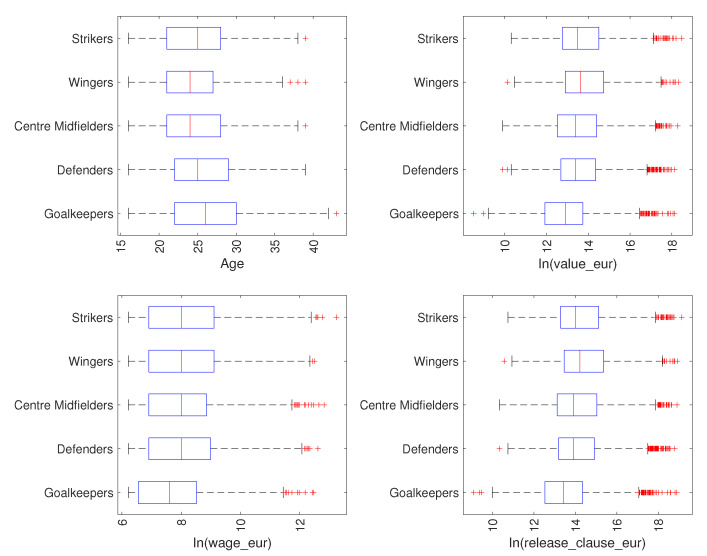
Box plots characterizing the attributes of {Goalkeepers, Defenders, Centre Midfielders, Wingers, Strikers} in the FIFA 2021 dataset.

Figure 2 shows the attributes age, ln(value_eur), ln(wage_eur) and ln(release_clause_eur), using box plots, for players in the groups {Goalkeepers, Defenders, Centre Midfielders, Wingers, Strikers}. In each box, the central trace stands for the median, while the bottom and top edges give the 25 and 75 percentiles, respectively. Moreover, the whiskers span between the extreme data points, without the outliers, which are represented by the symbol ‘+’. We can see that, on average, the Goalkeepers are older than field players, which translates to having longer carriers, and have lower value, salary and release clause contracts. Moreover, in all positions, we have many outliers, especially in ln(value_eur) and ln(release_clause_eur), meaning that we have a number of exceptions to the mainstream, particularly for the higher values.

**Figure 3:**
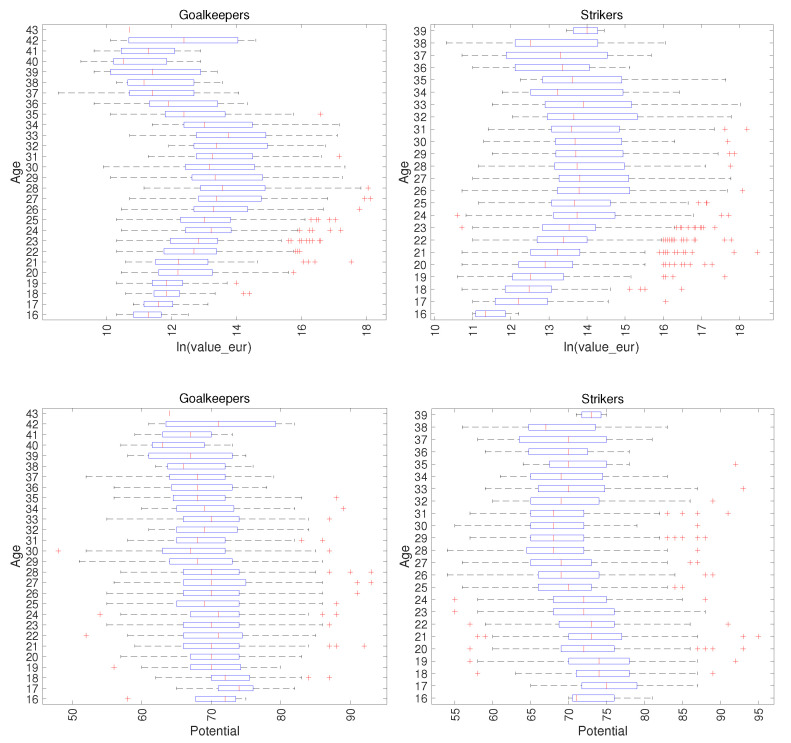
The attributes ln(value_eur) and potential versus age of Goalkeepers and Strikers (FIFA 2021 dataset).

In a different dimension, Figure 3 portrays the Goalkeeper’s and Striker’s attributes ln(value_eur) and potential versus age. We verify that for the attribute ln(value_eur), the Goalkeepers reach the maximum at the age of 27 and start losing value close to age 34 years old, respectively. For the Strikers, ln(value_eur) has its maximum at the age of 24 and then decreases smoothly. Regarding the attribute potential, for the Goalkeepers it diminishes slowly and monotonically since youth. For the Strikers, potential decreases until the age of 24, has a constant value up to the age of 31 and, then, surprisingly, it increases slightly almost until retirement.

**Figure 4:**
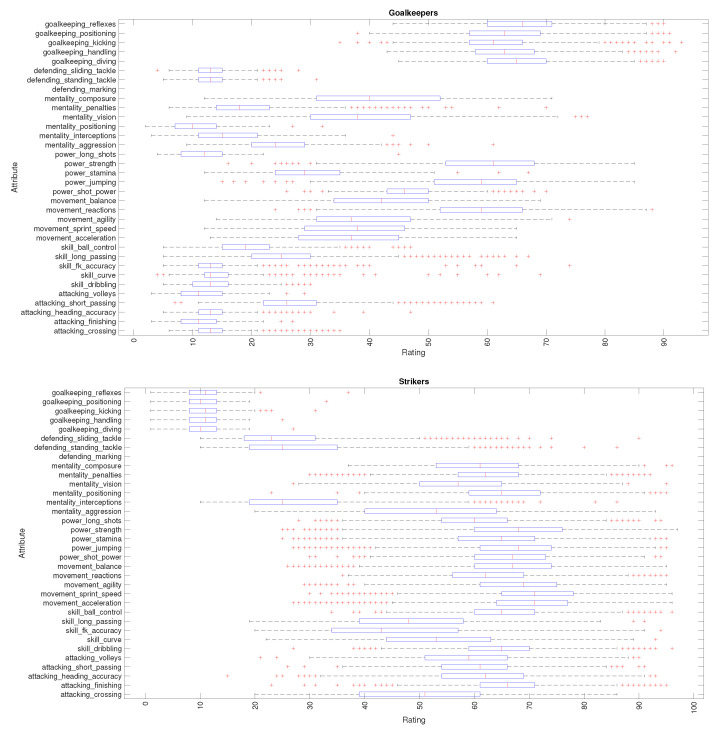
Attribute ratings of Goalkeepers and Strikers (FIFA 2021 dataset).

Figure 4 shows the attributes k=1,…,34 for Goalkeepers and Strikers. It should be mentioned that besides their ‘standard’ attributes, Goalkeepers and Strikers are also assigned with field player- and goalkeeper-specific attributes, respectively. This seems somewhat strange, but, in fact, soccer allows goalkeepers and field players to occupy any position on the pitch as long as they comply with the rules that apply to those positions. The analysis for other playing positions is not included here for the sake of parsimony.

## 4. The UMAP for Global Comparison and Visualization of Soccer Players

For implementing the UMAP dimensionality reduction, clustering and visualization tool we used the Matlab UMAP code, version 2.1.3, developed by Stephen Meehan et al. [67]. The function run_umap was called with the parameters n_neighbors and min_dist set to the values 10 and 0.2, respectively, adjusted by trial and error in order to obtain good visualization. These parameters correspond directly to *k* and δ introduced in Section 3. All other parameters were set to their default values.

We present results for the distances {Arccosine, Canberra, Correlation, Lorentzian} =$\left\{d^{Ar},d^{Ca},d^{Co},d^{Lo}\right\}$ to compare the objects $v_{i}$ and $v_{j}$, i,j=1,…,N, that stand for players and are characterized by the r=34 attributes (k=1,…,34) listed in Table 2. The choice for r=34 is based on the available database information. We included all players’ technical attributes (i.e., the maximum possible). The distances are given by [68]:

(10) $$d^{Ar}\left(v_{i},v_{j}\right)=\arccos(\frac{\sum_{k=1}^{r}v_{ik}\cdot v_{jk}}{\sqrt{\sum_{k=1}^{r}v_{ik}^{2}}\sqrt{\sum_{k=1}^{r}v_{jk}^{2}}}),$$

(11) $$d^{Ca}\left(v_{i},v_{j}\right)=\sum_{k=1}^{r}\frac{|v_{ik}-v_{jk}|}{|v_{ik}\left|+\right|v_{jk}|},$$

(12) $$d^{Co}\left(v_{i},v_{j}\right)={\left(1-\frac{\sum_{k=1}^{r}\left[v_{ik}-\mathrm{av}\left(v_{i}\right)\right]\left[v_{jk}-\mathrm{av}\left(v_{j}\right)\right]}{\sqrt{\sum_{k=1}^{r}{\left[v_{ik}-\mathrm{av}\left(v_{i}\right)\right]}^{2}}\sqrt{\sum_{k=1}^{r}{\left[v_{jk}-\mathrm{av}\left(v_{j}\right)\right]}^{2}}}\right)}^{\frac{1}{2}},$$

(13) $$d^{Lo}\left(v_{i},v_{j}\right)=\sum_{k=1}^{r}\ln(1+|v_{ik}-v_{jk}|).$$

**Figure 5:**
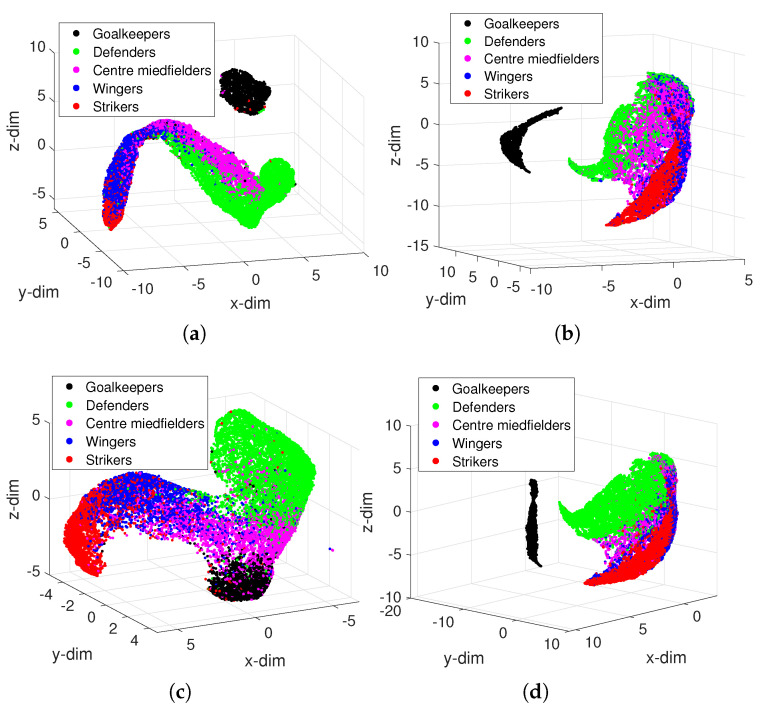
The 3D loci of players in the FIFA 2021 dataset obtained by the UMAP with the distances: (**a**) $d^{Ar}$; (**b**) $d^{Ca}$; (**c**) $d^{Co}$; (**d**) $d^{Lo}$.

Figure 5 depicts the 3D loci of the N=18,708 players in the FIFA 2021 dataset obtained by the UMAP with the distances $\left\{d^{Ar},d^{Ca},d^{Co},d^{Lo}\right\}$. We verified that the Goalkeepers form a cluster quite different from the others, while the {Defenders, Centre Midfielders, Wingers, Strikers} show some superposition. This is expected, since the field players have characteristics much different than those exhibited by the goalkeepers, but closer to each other. Moreover, we find players that have skills allowing them to play in different positions on the pitch. For example, L. Messi can play as RW, ST and CF. We verify also that the $d^{Ar}$, $d^{Ca}$ and $d^{Lo}$ separate well the five groups, while $d^{Co}$ reveals more difficulties to separate the Goalkeepers from the other groups. The $d^{Ca}$ and $d^{Lo}$ yield very similar loci.

Different distances can lead to valid visual representations, but not all of them are able to capture the structures of interest hidden in the data. It should be mentioned that the selection of an adequate distance often requires a number of numerical trials. In this work, we tested other distances, but the option of including additional metrics would have led to a huge number of figures. Therefore, we selected those that we found best, in order to limit space.

**Figure 6:**
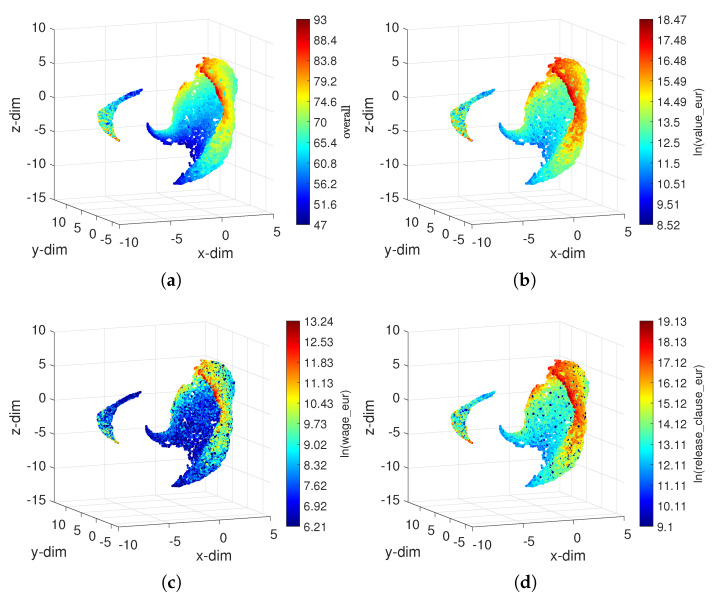
The 3D loci obtained by the UMAP with the Canberra distance $d^{Ca}$ for the FIFA 2021 dataset. The colormap is proportional to the attributes: (**a**) ln(overall); (**b**) ln(value_eur); (**c**) ln(wage_eur); (**d**) ln(release_clause_eur).

We can obtain an alternative representation by changing the fourth dimension from a categorical to a numerical variable. Figure 6 highlights different aspects of the 2021 dataset by means of colormaps applied to the locus obtained with $d^{Ca}$ proportional to the attributes ln(overall), ln(value_eur), ln(wage_eur) and ln(release_clause_eur). It can be seen that for all attributes, the UMAP can place similar objects close to each other in the embedding space. Moreover, the objects tend to distribute uniformly over a smooth surface. Naturally, other attributes can be represented using a similar procedure.

**Figure 7:**
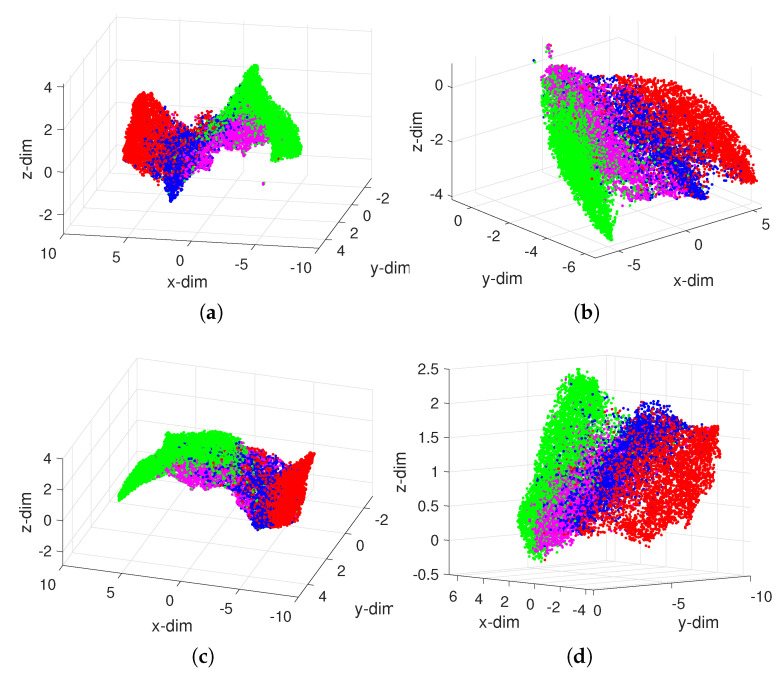
The 3D loci of players in the groups {Defenders, Centre Midfielders, Wingers, Strikers} the FIFA 2021 dataset obtained by the UMAP with the distances: (**a**) $d^{Ar}$; (**b**) $d^{Ca}$; (**c**) $d^{Co}$; (**d**) $d^{Lo}$.

It should be emphasized that we can compare subsets of players that are selected from the original dataset by means of some criterion. Figure 7 illustrates this idea by considering merely the players in the four groups {Defenders, Centre Midfielders, Wingers, Strikers}. In this case, the Goalkeepers were not included in the processed dataset, since, as shown in Figure 5, they are quite different from the others. We verify that now the four groups emerge slightly more clear than before, even though we still have some superposition.

## 5. The UMAP for Local Comparison and Visualization of Soccer Players

In this section, we analyze the UMAP loci for each group separately. In other words, we considered each group in the set {Goalkeepers, Defenders, Centre Midfielders, Wingers, Strikers} and, therefore, we have five cases. Obviously, the study can also be performed for other groups, for samples extracted from a single or various groups, and for distinct years.

**Figure 8:**
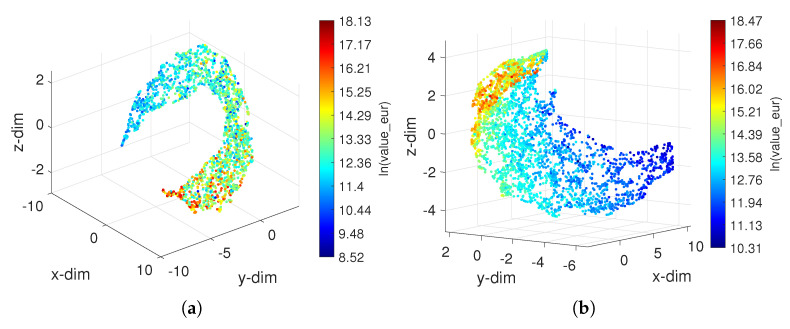
The 3D loci obtained by the UMAP with the Canberra distance for the FIFA 2021 dataset: (**a**) Goalkeepers; (**b**) Strikers. The colormap is proportional to the attribute ln(value_eur).

Figure 8 depicts the results obtained for Goalkeepers and Strikers, where the colormap is proportional to the attribute ln(value_eur). For the other groups, the charts are of the same type. We verify that, for both cases, the players, represented by points, distribute regularly in space, with the most valuable ones occupying the edges of the surface. Other possible patterns (if they exist) are difficult to distinguish due to the large number of objects and, thus, hide more subtle relationships. Therefore, even adopting 3D loci, to perceive assertively the location of the objects poses problems for a large number of objects. Magnifying the cloud of points mitigates the problem, but does not solve it satisfactorily. One possibility is to consider subsets with just the objects of interest and generate new (different) loci based on the the new datasets.

In the sequel, we analyze just the top 100 players in view of the criterion value_eur, in each group {Goalkeepers, Defenders, Centre Midfielders, Wingers, Strikers}. Naturally, other criteria can be adopted to extract the elements from the groups and we can mix players from various groups, but the criteria adopted illustrate well the procedure.

Firstly, the players are compared using the Canberra distance and their locus is generated through the UMAP dimensionality reduction and clustering algorithm. Secondly, given one element in the locus, freely chosen by the user, the *w* players who are closer to the one adopted as reference are identified according to the Euclidean distance in the 3D embedding space, yielding a small cluster of *w* elements. Finally, the user can evaluate the *w* most ‘interesting’ players in the perspective of additional criteria, such as value_eur, wage_eur or release_clause_eur. Of course, if w=1, then we have the player closer to the reference one.

**Figure 9:**
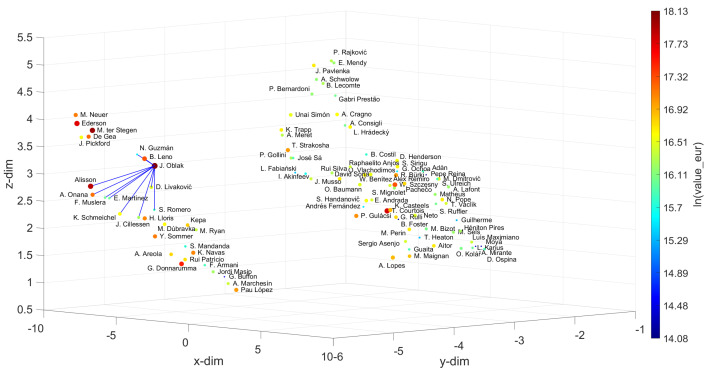
The 3D locus generated by the UMAP with the Canberra distance for the N=100 most valuable goalkeepers in the FIFA 2021 dataset. The reference is J. Oblak and w=10. The size of the circular marks and the colormap are proportional to the attributes wage_eur and value_eur, respectively.

**Figure 10:**
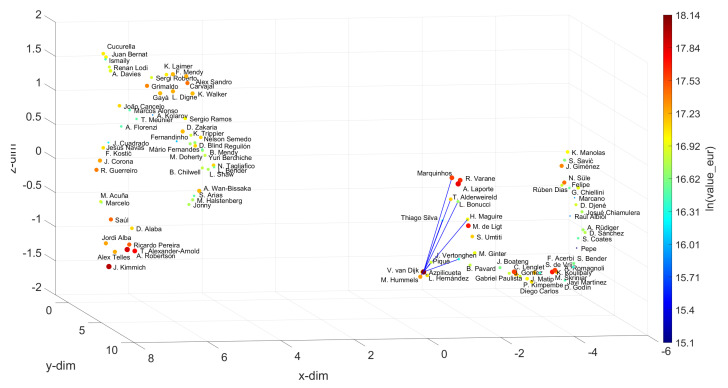
The 3D locus generated by the UMAP with the Canberra distance for the N=100 most valuable defenders in the FIFA 2021 dataset. The reference is V. van Dijkand and w=10. The size of the circular marks and the colormap are proportional to the attributes wage_eur and value_eur, respectively.

**Figure 11:**
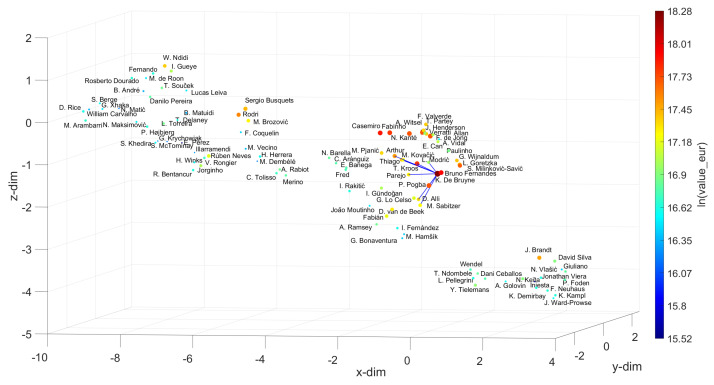
The 3D locus generated by the UMAP with the Canberra distance for the N=100 most valuable midfielders in the FIFA 2021 dataset. The reference is K. De Bruyne and w=10. The size of the circular marks and the colormap are proportional to the attributes wage_eur and value_eur, respectively.

**Figure 12:**
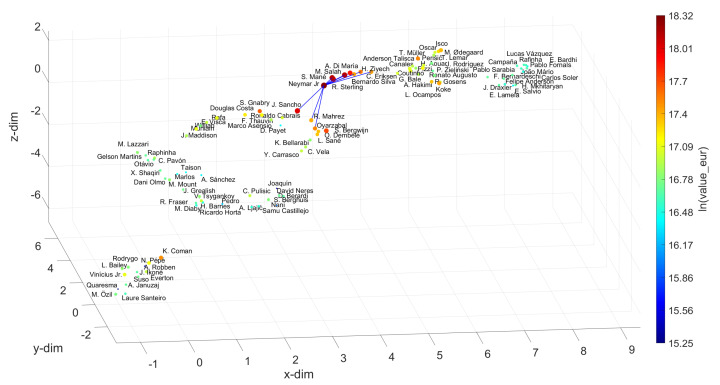
The 3D locus generated by the UMAP with the Canberra distance for the N=100 most valuable wingers in the FIFA 2021 dataset. The reference is Neymar Jr and w=10. The size of the circular marks and the colormap are proportional to the attributes wage_eur and value_eur, respectively.

**Figure 13:**
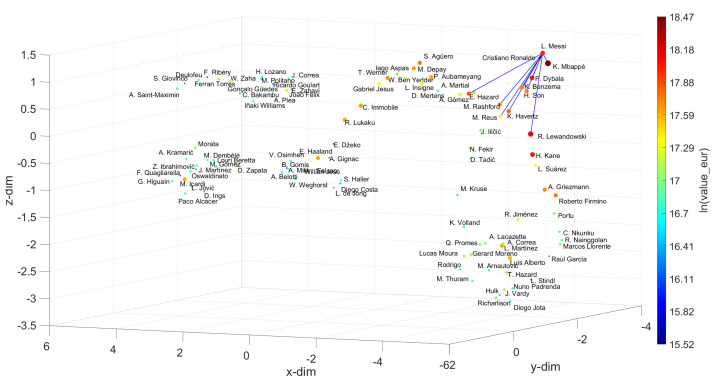
The 3D locus generated by the UMAP with the Canberra distance for the N=100 most valuable strikers in the FIFA 2021 dataset. The reference is L. Messi and w=10. The size of the circular marks and the colormap are proportional to the attributes wage_eur and value_eur, respectively.

Figure 9, Figure 10, Figure 11, Figure 12 and Figure 13 depict the UMAP loci generated. For the Goalkeepers, the most valuable one, J. Oblak, was taken as the reference. Then, choosing w=10, the closer elements, sorted by increasing distance, were {B. Leno, N. Guzmán, D. Livaković, S. Romero, E. Martínez, F. Muslera, K. Schmeichel, Alisson, A. Onana, J. Cillessen}. Therefore, B. Leno emerges as the best choice for substituting J. Oblak, when merely the player’s skills criterion is considered. However, if the user decides to choose additional criteria, such as value_eur and wage_eur, then a compromise exists between skills and cost, and the best choices could instead correspond to N. Guzmán or S. Romero, since they can be hired with a more limited economic effort.

For the Defenders, Centre Midfielders, Wingers and Strikers, we chose V. van Dijk, K. De Bruyne, Neymar Jr and L. Messi as references, and for w=10, we obtain the sets {M. Hummels, Piqué, Azpilicueta, L. Hernández, Thiago Silva, T. Alderweireld, J. Vertonghen, L. Bonucci, H. Maguire, Marquinhos}, {Bruno Fernandes, P. Pogba, L. Modrić, T. Kroos, D. Alli, Parejo, M. Kovačić, M. Sabitzer, Arthur, Thiago}, {S. Mané, R. Sterling, M. Salah, Bernardo Silva, A. Di María, H. Ziyech, J. Sancho, C. Eriksen, R. Mahrez, Oyarzabal} and {Cristiano Ronaldo, K. Mbappé, P. Dybala, K. Benzema, H. Son, K. Havertz, M. Rashford, M. Reus, R. Lewandowski, E. Hazard}, respectively. By applying the same approach as before for the Goalkeepers, the best options for substituting the references can be found. Let us focus on the Strikers. Usually, those are the most valuable and the most popular, as they are the most effective goal scorers, and goals are the essence of soccer. Let us assume that the recent conflicts between L. Messi and F. C. Barcelona of Summer 2020 have intensified and that the club is forced to replace the player. The question that will then be asked is whom to hire. According to the UMAP loci generated, the first choice will be Cristiano Ronaldo, if the criterion is exclusively based on the player’s skill. However, if there are no economic restrictions, as seems to be the case with elite clubs, the K. Mbappé hypothesis may be a more suitable choice. His value is higher and he earns a higher salary, but, on the other hand, he is younger and has greater potential for progression than Cristiano Ronaldo. Thus, it is up to the club to weigh the most convenient factors in deciding who should replace L. Messi.

**Figure 14:**
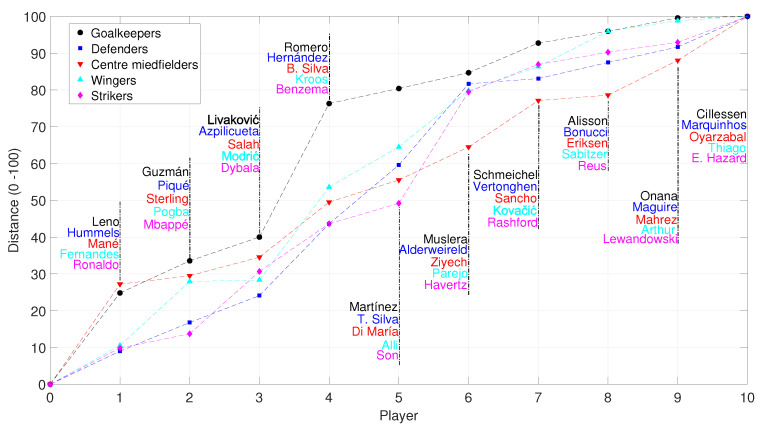
The normalized distance between the most valuable player in each group {Goalkeepers, Defenders, Centre Midfielders, Wingers, Strikers}, with reference {J. Oblak, V. van Dijk, K. De Bruyne, Neymar Jr, L. Messi}, and with relation to their j=1,…,10 closer elements.

Figure 14 portraits the normalized distance between the most valuable player in each group {Goalkeepers, Defenders, Centre Midfielders, Wingers, Strikers}, that is, having for references {J. Oblak, V. van Dijk, K. De Bruyne, Neymar Jr, L. Messi}, and comparing the UMAP coordinates with relation to their j=1,…,10 closer elements. We verify that the distance increases with jumps, which translate in worse skills as we move from first towards next choice players.

The UMAP was proven very effective for visualizing clusters of objects, outperforming other dimensionality reduction, clustering and information visualization techniques both in terms of their computational time, memory requirements and ability to unveil patterns embedded in the data [57]. One must note that concrete information about the management decisions of the soccer teams is not available. Therefore, to have a comparison of “real-world” data is virtually impossible, not only for researchers, but also for governments and for soccer associations. The experience gathered in other applications [69,70] allows us to consider whether a given algorithm is “better” or “worse” based on its clustering performance. Certainly, this is a subjective point of view, but the fact is that the assessment of the results provided by such kinds of techniques is based on the user experience and intuition. Another issue that needs to be highlighted is that the main goal of the paper is not to straightforwardly provide a commercial/computational tool for sport managers. Therefore, to avoid unclear legal, commercial, financial and ethical issues, the maximum extent for us was limited to refer the names of the players without commenting on their qualities. In summary, the goal of the paper is to explore the potential associated with the adoption of advanced clustering techniques for soccer players.

## 6. Conclusions

This paper adopted the UMAP dimensionality reduction, clustering and information visualization technique to explore relationships between soccer players. The algorithm constructs representations of the original dataset of players’ skills without imposing a priori requirements. The loci generated in a low-dimensional space allow a straightforward interpretation of the data. The results showed that the adoption of dimensionality-reduction and visualization tools for processing complex data is a key modeling option with current computational resources. The approach can be easily extended to deal with more features and richer descriptions of the data involving a higher number of dimensions.

## Figures and Tables

**Table 1:** List of typical positions of the players on the pitch and the number of players assigned to these positions in FIFA 2021 (April).

Group	Number of Players	Position	Acronym
Goalkeepers	2054	Goalkeepers	GK
Defenders	6725	Centre Back	CB
		Right Back	RB
		Left Back	LB
		Right Wing Back	RWB
		Left Wing Back	LWB
Centre Midfielders	3556	Centre Defensive Midfielder	CDM
		Centre Midfielder	CM
		Centre Attacking Midfielder	CAM
Wingers	2854	Right Midfielder	RM
		Left Midfielder	LM
		Right Wing	RW
		Left Wing	LW
Strikers	3519	Right Forward	RF
		Centre Forward	CF
		Left Forward	LF
		Striker	ST

**Table 2:** List of attributes of L. Messi and Cristiano Ronaldo in FIFA 2021 (April).

Atributes							
**Number**	**Name**	**Value**		**Number**	**Name**	**Value**	
k		**L. Messi**	**C. Ronaldo**	k		**L. Messi**	**C. Ronaldo**
1	attacking_crossing	85	84	26	mentality_composure	96	95
2	attacking_finishing	95	95	27	defending_marking	32	28
3	attacking_heading_accuracy	70	90	28	defending_standing_tackle	35	32
4	attacking_short_passing	91	82	29	defending_sliding_tackle	24	24
5	attacking_volleys	88	86	30	goalkeeping_diving	6	7
6	skill_dribbling	96	88	31	goalkeeping_handling	11	11
7	skill_curve	93	81	32	goalkeeping_kicking	15	15
8	skill_fk_accuracy	94	76	33	goalkeeping_positioning	14	14
9	skill_long_passing	91	77	34	goalkeeping_reflexes	8	11
10	skill_ball_control	96	92	35	sofifa_id	158023	20801
11	movement_acceleration	91	87	36	short_name	L. Messi	Cristiano Ronaldo
12	movement_sprint_speed	80	91	37	age	33	35
13	movement_agility	91	87	38	overall	93	92
14	movement_reactions	94	95	39	potential	93	92
15	movement_balance	95	71	40	value_eur	103.5 M	63M
16	power_shot_power	86	94	41	wage_eur	560 k	220k
17	powerjumping	68	95	42	player_positions	RW, ST, CF	ST, LW
18	power_stamina	72	84	43	release_clause_eur	212.2 M	104M
19	power_strength	69	78	44	height_cm	170	187
20	power_long_shots	94	93	45	weight_kg	72	83
21	mentality_aggression	44	63	46	preferred_foot	left	right
22	mentality_interceptions	40	29	47	international_reputation	5 (maximum 5)	5 (maximum 5)
23	mentality_positioning	93	95	48	work_rate	medium/low	high/low
24	mentality_vision	95	82	49	weak_foot	4 (maximum 5)	4 (maximum 5)
25	mentality_penalties	75	84	50	team_position	CAM	LS

## Data Availability

Data supporting reported results can be found at https://www.kaggle.com/stefanoleone992/fifa-21-complete-player-dataset (accessed on 12 February 2021).

## References

[1] Carling C., Williams A.M., Reilly T. (2007). Handbook of Soccer Match Analysis: A Systematic Approach to Improving Performance.

[2] Giulianotti R. (2012). Football. The Wiley-Blackwell Encyclopedia of Globalization.

[3] Couceiro M.S., Clemente F.M., Martins F.M., Machado J.A.T. (2014). Dynamical stability and predictability of football players: The study of one match. Entropy.

[4] Verstraete K., Decroos T., Coussement B., Vannieuwenhoven N., Davis J. (2019). Analyzing Soccer Players’ Skill Ratings Over Time Using Tensor-Based Methods. Joint European Conference on Machine Learning and Knowledge Discovery in Databases.

[5] Barron D., Ball G., Robins M., Sunderland C. (2018). Artificial neural networks and player recruitment in professional soccer. PLoS ONE.

[6] Folgado H., Duarte R., Fernandes O., Sampaio J. (2014). Competing with lower level opponents decreases intra-team movement synchronization and time-motion demands during pre-season soccer matches. PLoS ONE.

[7] Araújo D., Passos P., Esteves P., Duarte R., Lopes J., Hristovski R., Davids K. (2015). The micro-macro link in understanding sport tactical behaviours: Integrating information and action at different levels of system analysis in sport. Mov. Sport Sci.-Sci. Mot..

[8] Caetano F.G., da Silva V.P., da Silva Torres R., de Oliveira Anido R., Cunha S.A., Moura F.A. (2019). Analysis of match dynamics of different soccer competition levels based on the player dyads. J. Hum. Kinet..

[9] Neuman Y., Israeli N., Vilenchik D., Cohen Y. (2018). The adaptive behavior of a soccer team: An entropy-based analysis. Entropy.

[10] Merlin M., Cunha S.A., Moura F.A., Torres R.d.S., Gonçalves B., Sampaio J. (2020). Exploring the determinants of success in different clusters of ball possession sequences in soccer. Res. Sports Med..

[11] Ribeiro J., Davids K., Araújo D., Silva P., Ramos J., Lopes R., Garganta J. (2019). The role of hypernetworks as a multilevel methodology for modelling and understanding dynamics of team sports performance. Sports Med..

[12] Silva P., Duarte R., Esteves P., Travassos B., Vilar L. (2016). Application of entropy measures to analysis of performance in team sports. Int. J. Perform. Anal. Sport.

[13] Machado J.T., Lopes A.M. (2017). Multidimensional scaling analysis of soccer dynamics. Appl. Math. Model..

[14] Lopes A.M., Tenreiro Machado J. (2019). Entropy Analysis of Soccer Dynamics. Entropy.

[15] Lopes A.M., Tenreiro Machado J.A. (2020). Fractional Dynamics in Soccer Leagues. Symmetry.

[16] Berrar D., Lopes P., Davis J., Dubitzky W. (2019). Guest editorial: Special issue on machine learning for soccer. Mach. Learn..

[17] Karlis D., Ntzoufras I. (2003). Analysis of sports data by using bivariate Poisson models. J. R. Stat. Soc..

[18] Baio G., Blangiardo M. (2010). Bayesian hierarchical model for the prediction of football results. J. Appl. Stat..

[19] Hvattum L.M., Arntzen H. (2010). Using ELO ratings for match result prediction in association football. Int. J. Forecast..

[20] Berrar D., Lopes P., Dubitzky W. (2019). Incorporating domain knowledge in machine learning for soccer outcome prediction. Mach. Learn..

[21] Hubáček O., Šourek G., Železnỳ F. (2019). Learning to predict soccer results from relational data with gradient boosted trees. Mach. Learn..

[22] Tsokos A., Narayanan S., Kosmidis I., Baio G., Cucuringu M., Whitaker G., Király F. (2019). Modeling outcomes of soccer matches. Mach. Learn..

[23] Dobson S., Goddard J.A., Dobson S. (2001). The Economics of Football.

[24] Groot L. (2008). Economics, Uncertainty and European Football: Trends in Competitive Balance.

[25] Criado R., García E., Pedroche F., Romance M. (2013). A new method for comparing rankings through complex networks: Model and analysis of competitiveness of major European soccer leagues. Chaos Interdiscip. J. Nonlinear Sci..

[26] Pawlowski T., Breuer C., Hovemann A. (2010). Top clubs’ performance and the competitive situation in European domestic football competitions. J. Sports Econ..

[27] Dejonghe T., Van Opstal W. (2010). Competitive balance between national leagues in European football after the Bosman case. Riv. Dirit. Econ. Dello Sport.

[28] Liu G., Luo Y., Schulte O., Kharrat T. (2020). Deep soccer analytics: Learning an action-value function for evaluating soccer players. Data Min. Knowl. Discov..

[29] Link D. (2018). Data Analytics in Professional Soccer.

[30] Sellitto C., Hawking P. (2015). Enterprise systems and data analytics: A fantasy football case study. Int. J. Enterp. Inf. Syst. (IJEIS).

[31] Sha L., Lucey P., Zheng S., Kim T., Yue Y., Sridharan S. (2017). Fine-grained retrieval of sports plays using tree-based alignment of trajectories. arXiv.

[32] Tian C., De Silva V., Caine M., Swanson S. (2020). Use of machine learning to automate the identification of basketball strategies using whole team player tracking data. Appl. Sci..

[33] Wei X., Lucey P., Morgan S., Sridharan S. Predicting shot locations in tennis using spatiotemporal data. Proceedings of the 2013 International Conference on Digital Image Computing: Techniques and Applications (DICTA).

[34] Fernandez-Navarro J., Fradua L., Zubillaga A., McRobert A.P. (2019). Evaluating the effectiveness of styles of play in elite soccer. Int. J. Sports Sci. Coach..

[35] Wu Y., Xie X., Wang J., Deng D., Liang H., Zhang H., Cheng S., Chen W. (2018). Forvizor: Visualizing spatio-temporal team formations in soccer. IEEE Trans. Vis. Comput. Graph..

[36] Williams A.M., Reilly T. (2000). Talent identification and development in soccer. J. Sports Sci..

[37] Bidaurrazaga-Letona I., Lekue J.A., Amado M., Santos-Concejero J., Gil S.M. (2014). Identifying talented young soccer players: Conditional, anthropometrical and physiological characteristics as predictors of performance. Rev. Int. Cienc. Deporte.

[38] Sarmento H., Marcelino R., Anguera M.T., CampaniÇo J., Matos N., LeitÃo J.C. (2014). Match analysis in football: A systematic review. J. Sports Sci..

[39] Soto-Valero C. (2017). A Gaussian mixture clustering model for characterizing football players using the EA Sports’ FIFA video game system. Rev. Int. Cienc. Deporte.

[40] Strnad D., Nerat A., Kohek Š. (2017). Neural network models for group behavior prediction: A case of soccer match attendance. Neural Comput. Appl..

[41] Arndt C., Brefeld U. (2016). Predicting the future performance of soccer players. Stat. Anal. Data Min. ASA Data Sci. J..

[42] Rossi A., Pappalardo L., Cintia P., Iaia F.M., Fernàndez J., Medina D. (2018). Effective injury forecasting in soccer with GPS training data and machine learning. PLoS ONE.

[43] Moura F.A., Martins L.E.B., Cunha S.A. (2014). Analysis of football game-related statistics using multivariate techniques. J. Sports Sci..

[44] Brooks J., Kerr M., Guttag J. (2016). Using machine learning to draw inferences from pass location data in soccer. Stat. Anal. Data Min. ASA Data Sci. J..

[45] Louzada F., Maiorano A.C., Ara A. (2016). iSports: A web-oriented expert system for talent identification in soccer. Expert Syst. Appl..

[46] Maanijou R., Mirroshandel S.A. (2019). Introducing an expert system for prediction of soccer player ranking using ensemble learning. Neural Comput. Appl..

[47] Tenreiro Machado J., Lopes A.M., Galhano A.M. (2015). Multidimensional scaling visualization using parametric similarity indices. Entropy.

[48] Dunteman G.H. (1989). Principal Components Analysis.

[49] Thompson B. (2005). Canonical correlation analysis. Encyclopedia of Statistics in Behavioral Science.

[50] Tharwat A., Gaber T., Ibrahim A., Hassanien A.E. (2017). Linear discriminant analysis: A detailed tutorial. AI Commun..

[51] Child D. (1990). The Essentials of Factor Analysis.

[52] France S.L., Carroll J.D. (2010). Two-way multidimensional scaling: A review. IEEE Trans. Syst. Man Cybern. Part C.

[53] Lee J.A., Lendasse A., Verleysen M. (2004). Nonlinear projection with curvilinear distances: Isomap versus curvilinear distance analysis. Neurocomputing.

[54] Belkin M., Niyogi P. (2003). Laplacian eigenmaps for dimensionality reduction and data representation. Neural Comput..

[55] Coifman R.R., Lafon S. (2006). Diffusion maps. Appl. Comput. Harmon. Anal..

[56] Van der Maaten L., Hinton G. (2008). Visualizing data using t-SNE. J. Mach. Learn. Res.

[57] McInnes L., Healy J., Melville J. (2018). UMAP: Uniform manifold approximation and projection for dimension reduction. arXiv.

[58] Ware C. (2012). Information Visualization: Perception for Design.

[59] Spence R. (2001). Information Visualization: An Introduction.

[60] Abade E.A., Gonçalves B.V., Silva A.M., Leite N.M., Castagna C., Sampaio J.E. (2014). Classifying young soccer players by training performances. Percept. Mot. Ski..

[61] Fortuna F., Maturo F., Di Battista T. (2018). Clustering functional data streams: Unsupervised classification of soccer top players based on Google trends. Qual. Reliab. Eng. Int..

[62] Kirschstein T., Liebscher S. (2019). Assessing the market values of soccer players–a robust analysis of data from German 1. and 2. Bundesliga. J. Appl. Stat..

[63] Gavião L.O., Sant’Anna A.P., Alves Lima G.B., de Almada Garcia P.A. (2020). Evaluation of soccer players under the Moneyball concept. J. Sports Sci..

[64] Becht E., McInnes L., Healy J., Dutertre C.A., Kwok I.W., Ng L.G., Ginhoux F., Newell E.W. (2019). Dimensionality reduction for visualizing single-cell data using UMAP. Nat. Biotechnol..

[65] Dorrity M.W., Saunders L.M., Queitsch C., Fields S., Trapnell C. (2020). Dimensionality reduction by UMAP to visualize physical and genetic interactions. Nat. Commun..

[66] Cotta L., de Melo P., Benevenuto F., Loureiro A. (2016). Using Fifa Soccer Video Game Data for Soccer Analytics. https://homepages.dcc.ufmg.br/~fabricio/download/lssa_fifa_CR.pdf.

[67] Meehan C., Ebrahimian J., Moore W., Meehan S. (2021). Uniform Manifold Approximation and Projection (UMAP). https://www.mathworks.com/matlabcentral/fileexchange/71902.

[68] Deza M.M., Deza E. (2009). Encyclopedia of Distances.

[69] Machado J.T., Lopes A.M. (2020). Multidimensional scaling locus of memristor and fractional order elements. J. Adv. Res..

[70] Lopes A.M., Tenreiro Machado J.A. (2021). Dynamical Analysis of the Dow Jones Index Using Dimensionality Reduction and Visualization. Entropy.

